# Cardiac Myxomas Resembling Malignant Neoplasia: Incidentally Diagnosed vs. Cerebral Embolized Myxomas

**DOI:** 10.3390/cancers14051111

**Published:** 2022-02-22

**Authors:** Mohamed Salem, Jonas Hillmer, Christine Friedrich, Bernd Panholzer, Mohammed Saad, Mostafa Salem, Derk Frank, Markus Ernst, Walter Maetzler, Thomas Puehler, Georg Lutter, Felix Schoeneich, Assad Haneya, Jochen Cremer, Jan Schoettler

**Affiliations:** 1 Department of Cardiovascular Surgery, Campus Kiel, University Hospital Schleswig-Holstein, 24105 Kiel, Germany; jonas.hillmer@uksh.de (J.H.); christine.friedrich@uksh.de (C.F.); bernd.panholzer@uksh.de (B.P.); markus.ernst@uksh.de (M.E.); thomas.puehler@uksh.de (T.P.); georg.lutter@uksh.de (G.L.); felix.schoeneich@uksh.de (F.S.); assad.haneya@uksh.de (A.H.); jochen.cremer@uksh.de (J.C.); jan.schoettler@uksh.de (J.S.); 2 Department of Cardiology and Angiology, Campus Kiel, University Hospital Schleswig-Holstein, 24105 Kiel, Germany; mohammed.saad@uksh.de (M.S.); mostafa.salem@uksh.de (M.S.); derk.frank@uksh.de (D.F.); 3 DZHK (German Centre for Cardiovascular Research), Partner Site Hamburg/Kiel/Lübeck, Potsdamer Str. 58, 10785 Berlin, Germany; 4 Department of Neurology, Campus Kiel, University Hospital Schleswig-Holstein, 24105 Kiel, Germany; walter.maetzler@uksh.de

**Keywords:** cardiac tumors, myxomas, cerebral embolization

## Abstract

**Simple Summary:**

Cardiac tumors are relatively rare. Cardiac Myxomas (CM) are considered one of the most common benign cardiac tumors. They are usually distinct from soft tissue myxoma, most often occurring as a solitary, sporadic, pedunculated mass in the left atrium, and approximately 10% are associated with Carney syndrome with a high recurrence rate. They may cause sudden death, usually due to mitral valve obstruction or congestive heart failure. Malignant changes are known to occur but are extremely rare. They may mimic malignant neoplasia because of frequent embolism and can cause neurological deficits before theirsurgical removal. The current study analyzed the outcomes after operative myxoma excision in patients with or without cerebral embolic events prior to excision.

**Abstract:**

Background: Cardiac myxomas (CM) are the most common primary cardiac tumors in adults. They are usually benign; however, malignant changes are known to occur but are extremely rare. Embolization is a common complication of cardiac myxomas and can cause neurological deficits before their surgical removal. The current study analyzed the outcomes after operative myxoma excision in patients, with and without cerebral embolic events prior to excision. Methods: All 66 consecutive patients who underwent a surgical excision of CM between 2005 and 2019 at our department were analyzed retrospectively. Patients with (*n* = 14) and without (*n* = 52) preoperative strokes caused by cerebral tumor embolization were compared. Results: The mean age was 58.4 ± 12.7 years in the stroke group (SG) and 62.8 ± 11.7 years in the non-stroke group (N-SG) (*p* = 0.226). Gender (35.7% vs. 61.5% female; *p* = 0.084) did not differ significantly, and comorbidities were comparable in both groups. The left hemisphere in the territory of the middle cerebral artery was affected by preoperative cerebral infarction most commonly (28.6%). The time from diagnosis to cardiac surgery procedure was 7 (3–24) days in the SG and 23 (5–55) days in the N-SG (*p* = 0.120). Cardiac myxomas were localized in the left atrium in both groups more frequently (SG: 92.9% vs. N-SG: 78.8%; *p* = 0.436). In the SG, 57.1% of CM had a non-solid surface, were localized in the left heart, and had a pedunculated attachment away from the fossa ovalis. In the N-SG, 92.3% of CM did not meet all these criteria (*p* < 0.001). The maximal diameters of CM were comparable (SG: 3.4 ± 1.5 cm vs. N-SG: 3.8 ± 2.1 cm; *p* = 0.538). The operation times (192.5 (139.3–244.5) min vs. 215.5 (184.5–273.3) min; *p* = 0.046) and the cross-clamp times (54.5 (33.3–86.5) min vs. 78.5 (55–106.8) min; *p* = 0.035) were significantly shorter in the SG. Only in the N-SG were reconstructions of the endocardium with bovine pericardium required after resection (51.9% vs 0%; *p* < 0.001). In the N-SG, CM were explored via the right atrium more often (57.7% vs. 14.3%; *p* = 0.007). Patients in the N-SG required significantly shorter ICU care after surgery (*p* = 0.020). Other postoperative courses did not differ significantly. After tumor removal, 1.9% of the N-SG suffered their first stroke and 14.3% of the SG had a cerebral re-infarction (*p* = 0.111). The 30-day mortality rates were 1.9% in the N-SG and 7.1% in the SG (*p* = 0.382). In one case in the N-SG, a tumor recurrence was diagnosed. The Kaplan–Meiercurves showed a significantly better long-term prognosis for patients in the N-SG (*p* = 0.043). Conclusions: After the surgical removal of CM, the outcome is compromised if preoperative cerebral embolization occurs. Surgical treatment is therefore indicated as soon as possible, especially when CM have a non-solid surface, are localized in the left heart, and have a pedunculated attachment away from the fossa ovalis.

## 1. Introduction

Cardiac tumors are rare overall. The most common are cardiac myxomas (CM), which are usually localized in the left atrium [1,2,3]. Macroscopically, solid from non-solid or papillary CM are distinguished [4]. CM do not cause a specific sign of illness. Symptoms such as dyspnea, palpitations, chest pain, and syncope can occur [5]. When CM are not diagnosed incidentally, e.g., during echocardiography, cerebral emboli are often the first landmark clinical signs [6].

To prevent embolic complications, patients diagnosed with CM should be transferred to a cardiac surgery center immediately after diagnosis, where cardiac tumor extirpation can be performed promptly. Outcomes after the complete surgical excision of CM appear to be good, and recurrence rates are probably very low [7]. The bulk of publications on CM are case reports with impressive findings [3,8,9]. Only a few authors have published their general experience in a series of patients with CM in original papers.

It is uncertain to date whether the outcome differs in affected patients in whom CM was discovered incidentally or in whom the diagnosis was established after cerebral tumor embolization. On the basis of this background, the present article describes our experience over 15 years.

## 2. Materials and Methods

### 2.1. Patients

All patients who underwent extirpation of a CM between 1 January 2005 and 31 December 2019 at our Department of Cardiovascular Surgery were retrospectively analyzed. The cohort gavewritten informed consent for research with patient data. The study was approved by the local ethics committee (D518/20).

### 2.2. Diagnostics

**Figure 1:**
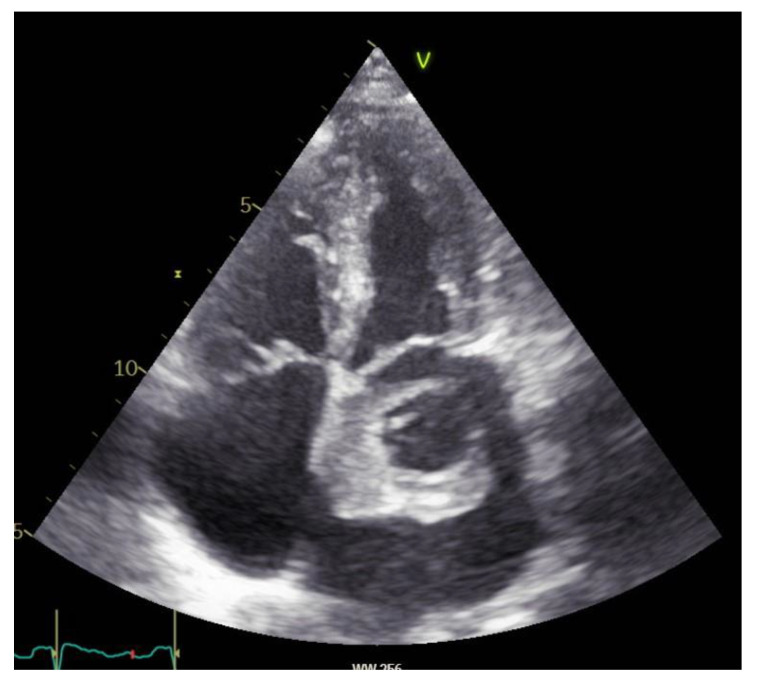
Echocardiographic imaging of a CM located in the left atrium.

**Figure 2:**
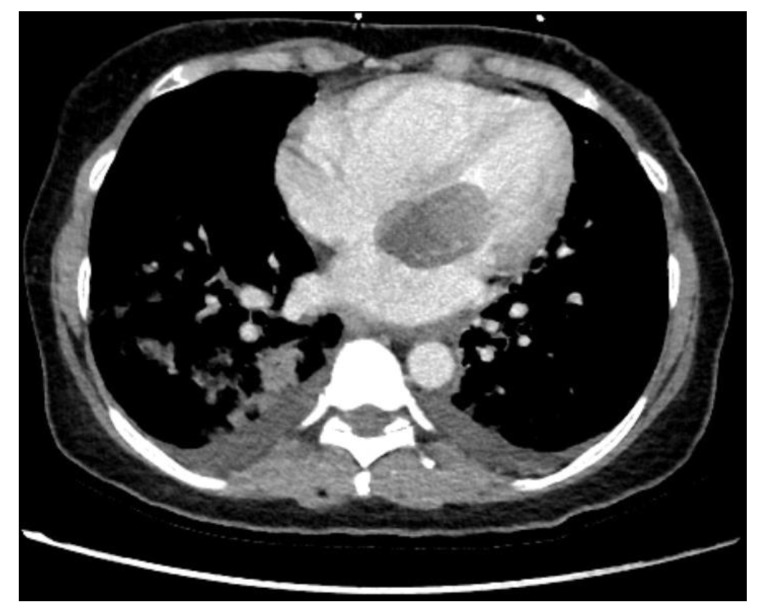
Visualization of a left atrial CM in a computed tomography scan with contrast medium.

**Figure 3:**
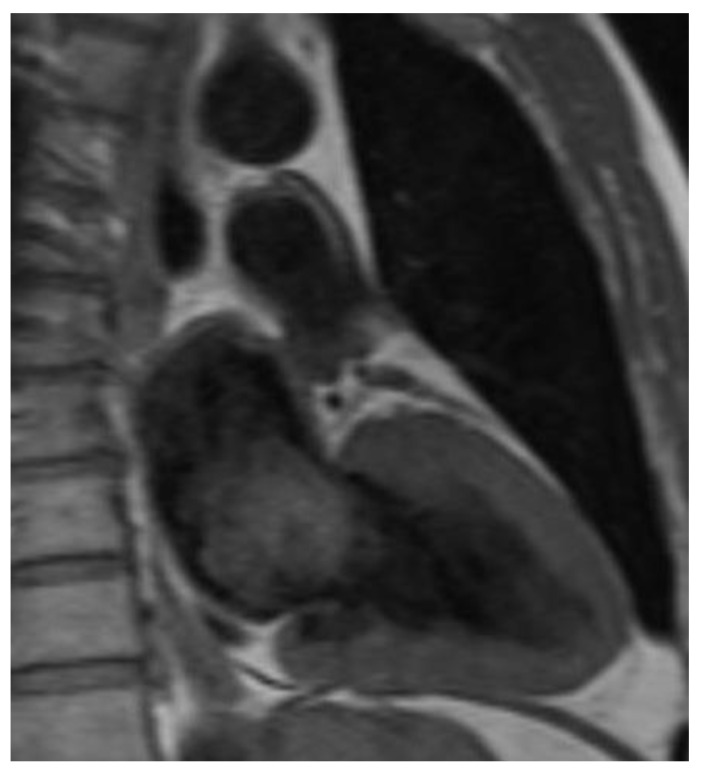
Magnetic resonance imaging of a left atrial CM.

All CM were diagnosed by echocardiography and/or computed tomography or magnetic resonance imaging (Figure 1, Figure 2 and Figure 3). After tumor excision, the diagnosis was confirmed by histological examination.

### 2.3. Surgical Techniques

**Figure 4:**
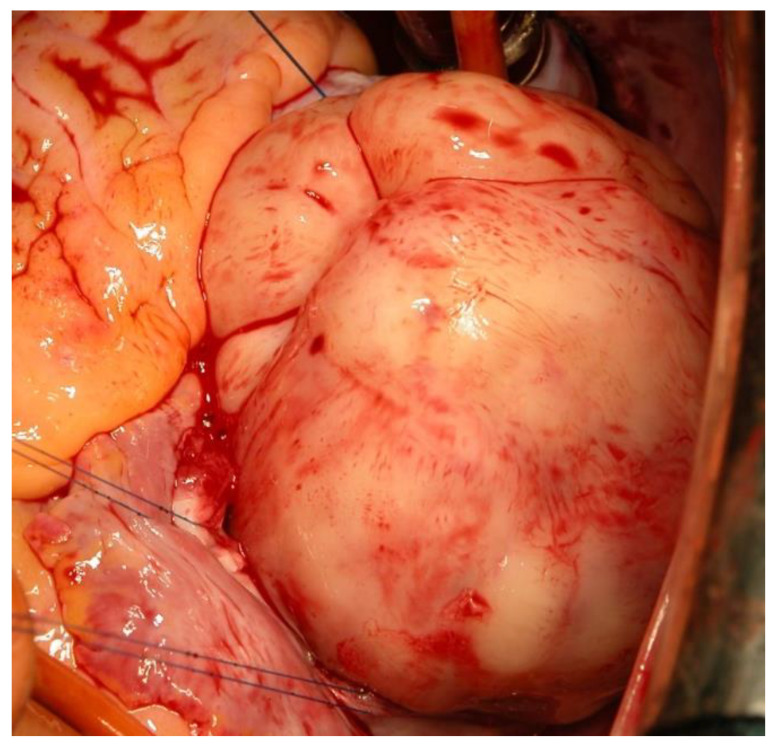
Intraoperative exploration of a CM via the right atrium.

After a median sternotomy, a heart–lung machine was connected via both the venae cavae and the ascending aorta. In cardioplegic cardiac arrest, the CM was then visualized via the right atrium, transseptally if necessary, and/or via the left atrium, depending on its location and extent (Figure 4). After the resection of the attachment site and complete tumor removal, bovine pericardium was used to cover the defects of the endocardium or atrial septum, if necessary. This was followed by the closure of the cardiac access pathways and necessary adjunctive cardiac surgery interventions.

In cases with a minimally invasive approach, the connection to the heart–lung machine was via the femoral artery and femoral vein, and, optionally, via the internal jugular vein. The heart was accessed via an anterolateral minithoracotomy in the 5th intercostal space on the right side. The CM was then salvaged in the same manner as conventional surgery, during a period of cardioplegic arrest.

### 2.4. Follow-Up

The follow-up was conductedby mail. If patients could not be reached, contact was made with the relevant registry offices.

### 2.5. Statistical Analysis

The statistical analysis was performed using the IBM SPSS Statistics for Windows (Version 28.0., Armonk, NY, USA). Characteristics of the patient groups were presented as mean and standard deviations and compared by unpaired t-tests for approximately normally distributed continuous variables, while not-normally distributed continuous data, as well as ordinal data, were presented as medians with 25th and 75th percentiles and compared by the Mann–Whitney U test. Normal distribution was assessed by the Kolmogorov–Smirnovtest. Categorial data were summarized as absolute (*n*) and relative (%) frequencies and compared by a Chi2test or Fisher’s exact test, as appropriate. Survival was calculated on right-censored data by Kaplan–Meier analyses and compared for differences between the groups by a log rank test. All tests were 2-sided and a *p*-value of ≤0.05 was regarded as statistically significant. The primary endpoint was 30-day mortality. Secondary endpoints were intraoperative variables, and postoperative courses (e.g., ventilation time, bleeding, acute renal failure, neurologic complications, and late mortality).

## 3. Results

A total of 66 consecutive patients underwent cardiac surgery for CM at our hospital between the beginning of 2005 and the end of 2019. Females accounted for 56.1% of patients and the mean age was 61.8 years.

A comparison of patients who had an embolic stroke related to CM before cardiac surgery (SG; *n* = 14) with those patients without prior stroke (N-SG; *n* = 52) showed no significant differences in preoperative patient characteristics, concomitant cardiac diseases, and diagnostic procedures. The majority of CM were diagnosed by echocardiography (Table 1, Table 2 and Table 3).

Regarding tumor-associated symptoms, neurological dysfunction was prominent in 100% of the SG. In 28.6% of patients, the left middle cerebral artery territory was affected. In the remaining patients (N-SG), dyspnea was the most common symptom in 34.6%, and 42.3% of them had no symptoms before diagnosis (Table 4).

The time from diagnosis to surgical removal of the CM did not differ significantly between the groups (7 (3–24) days in the SG and 23 (5–55) days in the N-SG; *p* = 0.120), and no patient suffered a first-time stroke or a recurrent stroke during the waiting period. Surgical time (215.5 min vs. 192.5 min; *p* = 0.046) and intraoperative clamping time (78.5 min vs. 54.5 min; *p* = 0.035) were significantly shorter in the SG. The respective proportion of minimally invasive approaches and combined cardiac surgery procedures did not differ significantly between the groups. CM access was significantly more frequent via the right atrium in the N-SG (57.7% vs. 14.3%; *p* = 0.007), and exclusively in the N-SG, the resection site on the endocardium was reconstructed using bovine pericardium in 51.9% of cases (*p* < 0.001) (Table 5).

**Figure 5:**
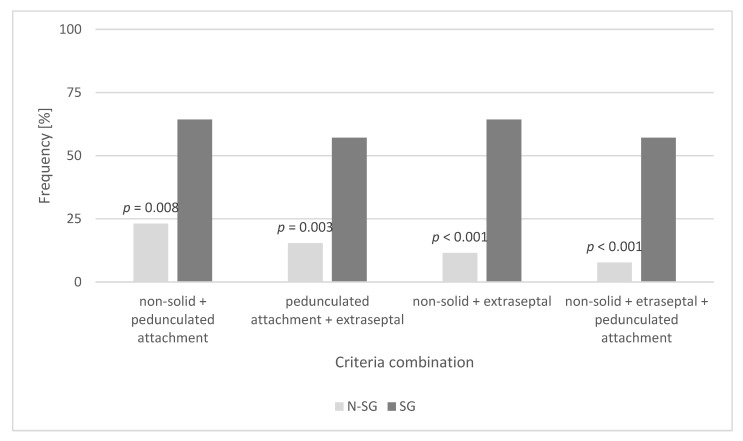
Criteria combination of CM in the N-SG vs. SG.

CM sizes of both groups were comparable (3.4 cm vs. 3.8 cm in their maximum lengths) (SG vs. N-SG; *p* = 0.538). CM location was not significantly different between both groups (*p* = 0.436); most CM (92.9% (SG) and 78.8% (N-SG)) were found in the left atrium. In the SG, 57.1% of CM had a non-solid surface, were localized in the left heart, and had a pedunculated attachment away from the fossa ovalis. In the N-SG, 92.3% of CM did not meet all these criteria (*p* < 0.001) (Table 6 and Figure 5).

Patients in the N-SG required a significantly shorter time in the intensive care unit after surgery (2 vs. 3 days; *p* = 0.020). No other significant differences during hospitalization were observed. In the early course after tumor removal, 1.9% of the N-SG suffered their first stroke, and 14.3% of the SG had a cerebral re-infarction (*p* = 0.111). The 30-daymortality was 1.9% (N-SG) and 7.1% (SG), with no significant discrepancy (*p* = 0.382). One year after operation, the mortality rates were unchanged (Table 7).

**Figure 6:**
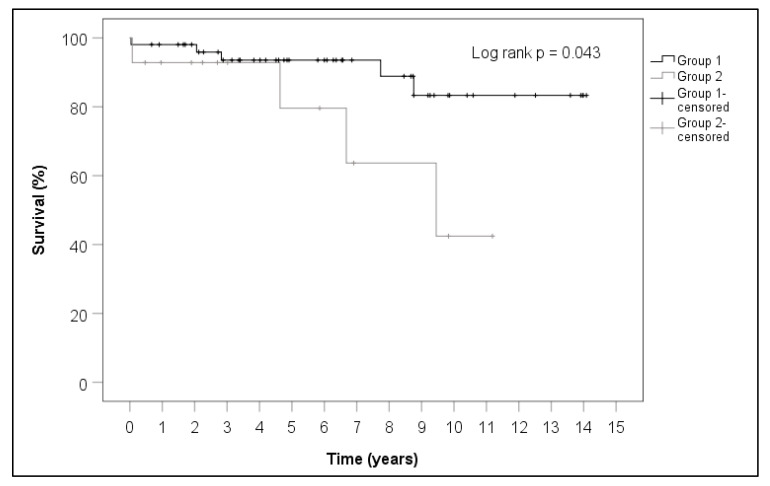
Kaplan–Meier curves of group 1 (N-SG) and group 2 (SG) with right-censored survival for both groups.

The follow-up showed that one patient in the N-SG underwent a re-operation for a recurrence of CM at our hospital. New strokes in the later course were not documented in eitherstudy group. Mortality was not significantly different at the time of follow-up (28.6% (SG) vs. 9.6% (N-SG); *p* = 0.087). The calculations for the Kaplan–Meier curve showed a significant survival advantage for the patients in the N-SG (*p* = 0.043) (Table 8 and Figure 6). Survival of the N-SG group and the SG group was 98% vs. 92% after one year, 93% vs. 92% after three years and 93% vs. 79% after five years.

## 4. Discussion

CM were represented in the literature through numerous case reports but in few original papers [3]. The aim of the present work was to investigate whether the outcomes of patients who underwent removal of CM with or without pre-excision stroke differ. Special attention was paid to CM-related characteristics associated with preoperative stroke.

During the 15-year period considered, a total of 66 consecutive patients, 14 with (SG) and 52 without previous stroke (N-SG), underwent cardiac surgery for CM at our hospital. Considering the low incidence of CM, estimated at 0.5 cases per 1 million individuals per year, the low number of assigned CM patients is not surprising, even in larger cardiac centers [7]. Females accounted for 56.1% of our studied patients. Other study groups were also more likely to encounter females suffering from CM than males. In the work of Tasoglu et al., as many as 77.6% of CM patients were female [8]. The ratio of cerebral embolized cardiac myxomas in our collective is similar to the observations of Stefanou et al., who found eight patients with embolic stroke among 52 CM patients in their clinic within 12 years [10].

CM localized in the left heart can potentially embolize in all cerebral areas. If a stroke was present preoperatively in our group, the left middle cerebral artery territory was most frequently affected (28.6%). Interestingly, Lee et al. also found a particularly frequent embolization of CM into the middle cerebral arteries in their studies [11]. Our patients in the SG disproportionately (57.1%) had CM that were non-solid and had a pedunculated site of attachment in the left heart away from the fossa ovalis. In N-SG, 92.3% of the diagnosed CM did not meet this combination of the above criteria. Keeling et al. found a significant reason for embolization in terms of tumor consistency in their study [12]. Swartz and coworkers observed a significant association between an extraseptal attachment site and neurologic events in their collective [13]. Our results did not provide a single criterion for assessing the risk of embolization. Only the combination of at least two of the criteria we mentioned was significantly associated with cerebral embolization in our collective. With regard to the risk of embolization, the size of the CM per se did not play a role in our evaluations. This was confirmed by Garatti and colleagues [14]. The significantly longer operative time and clamping time in the N-SG are related to the need for plastic reconstruction of the resection site with bovine pericardium. In our studies, no patients in the SG required such reconstruction, suggesting that there were no particularly deep or wide defects after the complete resection of the CM in this group. In the SG, the CM was explored less frequently via the right atrium, because it is almost localized in all patients in the left heart. Since 17.3% of the patients in N-SG had CM localization in the right atrium, it is plausible that exploration via the right atrium was significantly more frequent in these patients. We do not consider it unusual that patients with a previous stroke require a longer stay in the intensive care unit after cardiac surgery; moreover, the total hospital length of stay was comparable in both of our groups. Basically, cardiac surgery on the heart–lung machine with full heparinization in patients with acute or subacute stroke is associated with the risk of intracerebral hemorrhage during the procedure. Fortunately, however, none of our patients suffered this severe complication.

Most of our patients underwent regular cardiological examinations postoperatively. One patient (1.5%), who belonged to the N-SG, was diagnosed with a recurrence of CM, which required repeat surgical extirpation at our hospital. Other study groups reported similarly low recurrence rates [11,14,15]. Neurologic follow-up was utilized by only one of our patients. In both groups, patients who participated in the follow-up did not experience significantly different rates of new, clinically manifested neurologic events during the course after surgery. Although 1-year mortality was still not significantly different, our patients in SG had a significantly worse long-term prognosis; however, the prognosis was still better than those patients with other malignant cardiac sarcomas, where their prognosis remains very poor and is considered a lethal disease [16].

## 5. Conclusions

It can be concluded that the prognosis after the removal of CM by cardiac surgery is overall favorable. Although a previous stroke due to the embolization of tumor fragments usually does not severely compromise the affected patients neurologically, their life expectancy in the long-term course is significantly worse than in CM patients without preoperative cerebral embolization. Due to the preoperative embolic risk, patients with diagnosed CM should be treated urgently. Particular urgency seems to be required for CM that are non-solid with a pedunculated attachment in the left heart away from the fossa ovalis. A postoperative follow-up of the patients affected by preoperative stroke should be carried out on a regular interdisciplinary basis over a long period of time. Future studies need to show whether the preoperative neuroradiologic removal of cerebrally embolized tumor fragments may improve prognosis.

## 6. Limitations

The present work has the disadvantages associated with retrospective studies. The small number of patients is due to the fact that CM surgery is rare overall, even in large cardiac surgery centers. A 100% follow-up could only be achieved with regard to the mortality statistics. Because the deceased patients were not autopsied, no information on the cause of death can be provided.

## Figures and Tables

**Table 1:** Preoperative patient characteristics.

Preoperative Patient Characteristics	SG	N-SG	*p*-Value
Mean age (years)	58.4 ± 12.7	62.8 ± 11.7	0.226
Female gender (%)	35.7	61.5	0.084
Mean body mass index	27.2 ± 4.4	26.1 ± 4.3	0.408
Mean left ventricle ejection fraction (%)	70 (63–70)	65 (60–70)	0.113
Preoperative atrial fibrillation (%)	14.3	17.3	1.000
Pulmonary hypertension *(%)	14.3	32.7	0.318
Arterial hypertension (%)	50.0	53.8	0.798
Diabetes mellitus (%)	14.3	11.5	0.674
Hyperlipoproteinemia **	57.1	30.8	0.069
History of smoking (%)	42.9	55.8	0.390
Chronic obstructive lung disease (%)	7.1	7.7	1.000
Peripheral artery disease (%)	7.1	3.8	0.517
Oncologic disease (%)	7.1	3.8	0.517

*: > than 25 mmHg, **: total cholesterol > 240 mg/dL or low-density lipoprotein (LDL) > 160 mg/dL, SG: stroke group; N-SF: non-stroke group.

**Table 2:** Concomitant cardiac diseases.

Concomitant Cardiac Diseases	SG	N-SG	*p*-Value
Coronary artery disease (%)	42.9	36.5	0.665
Mitral valve insufficiency (%)	35.7	57.7	0.144
Persistent foramen ovale (%)	7.1	5.8	1.000
Atrial septal defect (%)	0	1.9	1.000

**Table 3:** Diagnostic methods.

Diagnostic Methods	SG	N-SG	*p*-Value
Transthoracal echocardiography (%)	57.1	73.1	0.328
Transesophageal echocardiography (%)	85.7	69.2	0.318
Magnetic resonance imaging (%)	7.1	13.5	1.000
Computertomography (%)	7.1	15.4	0.671
Left heart catheterization (%)	92.9	88.5	1.000

**Table 4:** Tumor-associated symptoms.

Tumor-Associated Symptoms	SG	N-SG	*p*-Value
Chest pain (%)	7.1	11.5	1.000
Palpitation (%)	0	9.6	0.576
Dyspnea (%)	0	34.6	0.007
Syncope (%)	7.1	9.6	1.000
Fatigue (%)	0	9.6	0.576
Neurological dysfunction (%)	100	0	<0.001
Asymptomatic (%)	0	42.3	0.003

**Table 5:** Intraoperative data.

Intraoperative Data	SG	N-SG	*p*-Value
Time from diagnosis to surgery (days)	7 (3;24)	23 (5;55)	0.120
Minimal invasive surgery (%)	28.6	42.3	0.350
Mean operating time (min)	192.5	215.5	0.046
Mean bypass time (min)	102.5 ± 45.7	133.1 ± 54.3	0.058
Mean aortic-cross-clamptime (min)	54.5	78.5	0.035
Endocardial reconstruction (%)	0	51.9	<0.001
Concomitant cardiac surgery (%)	21.4	34.6	0.520

**Table 6:** Details of extirpated cardiac myxomas.

Details of Extirpated Cardiac Myxomas	SG	N-SG	*p*-Value
Location in the left ventricle (%)	4.5	3.8	0.517
Location in the left atrium (%)	92.9	78.8	1.000
Location in the right atrium (%)	0	17.3	0.186
Atrial septum attachment (%)	21.4	50.0	0.056
Pedunculated tumor base (%)	51.5	46.2	0.093
Maximal tumor size (cm)	3.4 ± 1.5	3.8 ± 2.1	0.538
Non-solid tumor type (%)	78.6	50.0	0.103
Calcification of the tumor	7.1	13.5	1.000

**Table 7:** Postoperative clinical data.

Postoperative Clinical Data	SG	N-SG	*p*-Value
Stay on intensive care unit (d)	3	2	0.020
Ventilation time (h)	10.5	10.5	0.783
Tracheotomy (%)	7.1	5.8	1.000
Rethoracotomy (%)	0	3.8	1.000
Postoperative atrial fibrillation (%)	7.1	26.9	0.161
Postoperative stroke (%)	14.3	1.9	0.111
Postoperative dialysis (%)	7.1	1.9	0.382
Hospital stay (d)	11.0	12.5	0.776
Thirty-day mortality (%)	7.1	1.9	0.382

**Table 8:** Follow-up.

Follow-Up	SG	N-SG	*p*-Value
Rate of response (%)	42.9	55.8	0.390
Mortality (%)	28.6	9.6	0.087
Stroke after hospital discharge (%)	0	0	1.000
Recurrence of myxoma (%)	0	3.4	1.000
Cardiac redo surgery (%)	0	3.4	1.000
NYHA I-II (%)	50.0	27.6	0.352
Neurological limitations (%)	66.7	0	<0.001

## Data Availability

The data presented in this study are available on request from the corresponding author.

## References

[1] Reynen K. (1996). Frequency of primary tumors of the heart. Am. J. Cardiol..

[2] Pinede L., Duhaut P., Loire R. (2001). Clinical Presentation of Left Atrial Cardiac Myxoma. Medicine.

[3] Schiele S., Maurer S.J., Pujol Salvador C., Vitanova K., Weirich G., Meierhofer C., Voss B., Ewert P., Tutarel O. (2019). Left Atrial Myxoma: When Big Is Too Big. Circ. Cardiovasc. Imaging.

[4] Roeltgen D., Kidwell C.S. (2014). Neurologic complications of cardiac tumors. Handb. Clin. Neurol..

[5] Cetin G., Gursoy M., Ugurlucan M., Uzunhasan I., Hatemi A.C., Tireli E., Kucukoglu S., Kansiz E. (2010). Single-institutional 22 years experience on cardiac myxomas. Angiology.

[6] Grothusen C., Schöttler J., Cremer J., Frey N., Langer C. (2014). Very large left atrial myxoma: An unusual differential diagnosis of bronchial hyper-reactivity. Cardiovasc. Diagn. Ther..

[7] MacGowan S.W., Sidhy P., Aherne T., Luke D., Wood A.E., Neligan M.C., McGovern E. (1993). Atrial myxoma: National incidence, diagnosis and surgical management. Ir. J. Med. Sci..

[8] Tasoglu I., Tutun U., Lafci G., Hijaazi A., Yener U., Yalcinkaya A., Ulus T., Aksoyek A., Saritas A., Birincioglu L. (2009). Primary Cardiac Myxomas: Clinical Experience and Surgical Results in 67 Patients. J. Card. Surg..

[9] García-Quintana A., Martín-Lorenzo P., Suárez de Lezo J., Díaz-Escofet M., Llorens R., Medina A. (2005). Mixoma auricular izquierdo infectado [Infected left atrial myxoma]. Rev. Esp. Cardiol..

[10] Stefanou M.I., Rath D., Stadler V., Richter H., Hennersdorf F., Lausberg H.F., Lescan M., Greulich S., Poli S., Gawaz M.P. (2018). Cardiac Myxoma and Cerebrovscular Events: A Retrospective Cohort Study. Front. Neurol..

[11] Lee S.J., Kim J.H., Na C.Y., Oh S.S. (2012). Eleven Year’s Experience with Korean Cardiac Myxoma Patients: Focus on Embolic Complications. Cerebrovasc. Dis..

[12] Keeling I.M., Oberwalder P., Anelli-Monti M., Schuchlenz H., Demel U., Tilz G.P., Rehak P., Rigler B. (2002). Cardiac myxomas: 24 years of experience in 49 patients. Eur. J. Cardiothorac. Surg..

[13] Swarzt M.F., Lutz C.J., Chandan V.S., Landas S., Fink G.W. (2006). Atrial Myxomas: Pathologic Types, Tumor Location, and Presenting Symptoms. J. Cardiac. Surg..

[14] Garatti A., Nano G., Canziani A., Gagliardotto P., Mossuto E., Frigiola A., Menicanti L. (2012). Surgical Excision of Cardiac Myxomas: Twenty Years Experience at a Single Institution. Ann. Thorac. Surg..

[15] Khan M., Sanki P., Hossain M., Charles A., Bhattacharya S., Sarkar U. (2013). Cardiac myxoma: A surgical experience of 38 patients over 9 years, at SSKM hospital Kolkata, India. South Asian J. Cancer.

[16] Simpson L., Kumar S.K., Okuno S.H., Schaff H.V., Porrata L.F., Buckner J.C., Moynihan T.J. (2008). Malignant primary cardiac tumors: Review of a single institution experience. Cancer.

